# Prediction method of longitudinal surface settlement caused by double shield tunnelling based on deep learning

**DOI:** 10.1038/s41598-023-49096-z

**Published:** 2024-01-09

**Authors:** Wentao Shang, Yan Li, Huanwei Wei, Youbao Qiu, Chaowei Chen, Xiangrong Gao

**Affiliations:** 1 College of Civil Engineering, Shandong Jianzhu University, Jinan, 250101 People’s Republic of China; 2 Key Laboratory of Building Structural Retrofitting and Underground Space Engineering (Shandong Jianzhu University), Ministry of Education, Jinan, 250101 People’s Republic of China; 3 Shandong Jianzhu University Subway Protection Research Institute, Jinan, 250101 People’s Republic of China; 4 Shandong Hi-Speed Group Co., Ltd, Jinan, 250014 People’s Republic of China; 5 Shandong Jianhe Civil Engineering Consulting Co., Ltd, Jinan, 250013 People’s Republic of China

**Keywords:** Civil engineering, Computational science, Computer science

## Abstract

The deep learning method faces the challenges of small sample data and high dimensional shield operational parameters in predicting the longitudinal surface settlement caused by shield excavation. In this study, various optimization algorithms were compared, and the slime mould algorithm (SMA) was optimally chosen to optimize the hyperparameters of random forest (RF), and SMA-RF was used for dimensionality reduction and feature contribution analysis. A double-input deep neural network (D-DNN) framework was proposed for the prediction of surface settlement, which considers the influence of twin tunnels and effectively increases the high-fidelity data in the database. The results show that SMA performs best among various optimization algorithms; employing features that have a cumulative contribution value exceeding 90% as input can result in high prediction accuracy; there is significant uncertainty in the feature contribution analysis for small sample data; the reduced shield running parameters show a strong nonlinear relationship with surface settlement; compared with S-DNN, D-DNN takes into account the excavation of twin tunnels and expands the database capacity by more than 1.5 times, with an average increase of 27.85% in the R^2^ and an average decrease of 53.2% in the MAE.

## Introduction

Owing to the rapid development of underground infrastructure, the shield tunnelling method has become widely employed in geotechnical engineering. Shield excavation can induce surface settlement and significantly affects the surrounding environment^1,2^, thus potentially resulting in surface collapse and the tilting of surrounding structures. To safeguard the surrounding environment, one must accurately forecast surface settlement^3–5^.

Surface settlement caused by shield excavation is affected by various factors, including soil loss, erosion, stress release, and soil consolidation^6–8^. Conventional research methods include empirical formula approaches^9–11^, numerical simulation techniques^12–15^, and model testing methods^16–19^. However, when complex construction factors and geological conditions are involved, these methods yield results that deviate significantly from observed data. Machine learning methods^20–23^, particularly deep learning methods, are promising for predicting surface settlement as they consider multiple factors and consider the relationship between the input variables and surface settlement^24,25^.

Shield tunnelling is a dynamic and continuous process^26^. Before and after shield tunnelling, the surface generally undergoes a slight uplift, followed by gradual sinking. The development process of surface settlement must be analysed and predicted accurately to control surface settlement. When predicting the longitudinal surface settlement using deep learning methods, feature selection is critical because it directly affects the performance of data-driven models^27^. Zhang et al.^28^ and Li et al.^29^ compared the effects of different input features and discovered that selecting the appropriate features can effectively improve prediction accuracy. However, in existing studies, features from the entire dataset are rarely selected^30^, whereas only features with high correlation are selected, which may result in information loss and affect the prediction accuracy. Moreover, when encountering the same target task, the input features of the model should be selected based on the actual situation, because it is highly improbable for a single ML model to predict the behaviour of each rock mass accurately with equal precision by utilizing identical input variables^31^.

Studies pertaining to longitudinal surface settlement utilise settlement information per ring, thereby significantly increasing the database capacity compared with studies pertaining to the maximum surface settlement^26^. However, the high excavation speed of the shield tunnelling method causes inadequate monitoring of surface settlement, resulting in a limited database capacity compared to other machine learning problems^32,33^. Ye et al.^34^ obtained surface settlement data by tunnelling under ancient towers and compared the prediction accuracy of databases with different capacities. The authors indicated the possibility of inadequate training when the data volume was not sufficiently large. Table 1 demonstrates the common research methods currently used to tackle the problem of inadequate database data. These methods involve employing effective feature extraction techniques^35–37^, enhancing the database through the inclusion of outcomes derived from physical formulas^38,39^, numerical simulation results^40^, and results obtained using deep learning algorithms^41^. However, these methods do not contribute to the enrichment of high-fidelity data within their own database. Essentially, they primarily involve further exploration of their own database or the incorporation of low-fidelity data. Prior research has demonstrated that the availability of additional high-fidelity data can greatly enhance algorithmic model training^38^. Consequently, in practical engineering applications, the incorporation of high-fidelity data holds enormous potential for significantly improving prediction accuracy.

**Table 1:** Existing research on small sample data solutions.

Method	Literature	Application	Database research methods
CEEMDAN-LSTM	Cao et al.^35^	Tunnel Ground Surface Settlement Prediction	Database deep mining
Adaboost.RT framework	Yan et al.^37^	Tunnel Ground Surface Settlement Prediction	
GL-Net	Li et al.^36^	Semantic segmentation for point clouds of shield tunnel	
Empirical formulas	Chen and Feng^38^	Predicting the shear capacity of reinforced concrete	Supplement low-fidelity data
Physical model	Qiu et al.^39^	Fault diagnosis for shield Machine hydraulic system	
FE model	Fang et al.^40^	Assessment of end-two-flange web crippling strength	
WGAN-GP	Zhu et al.^41^	Tunnel Lining Defect Identification	

Complete ensemble empirical mode decomposition with the adaptive noise long short term memory (CEEMDAN-LSTM); Deep learning network for the point cloud segmentation task of shield tunnel scene (GL-Net); Deep belief network (DBN); Wasserstein generative adversarial network (WGAN-GP).

To shorten the construction period, numerous engineering projects employ the strategy of sequential excavation of two tunnels^42^, which collectively contribute to surface settlement. However, most existing studies primarily focus on the parameters of a single tunnel^43–45^, overlooking those of the other tunnel. This approach, despite its cost-saving implications, impacts calculation accuracy due to the potential for increased surface settlement resulting from the excavation and geotechnical actions in dual-line tunnels^46^. Thus, a thorough investigation of the shield tunnelling parameters, spatial positioning relationship, as well as soil parameters of dual-line tunnels in a methodical manner becomes imperative.

To summarize, the present research encounters the following challenges: (1) efficient and rational feature selection from an entire dataset have not been investigated sufficiently; (2) the capacity of a database for longitudinal settlement is relatively small, which can result in inadequate training; and (3) the combined effect of double-line tunnel excavation on surface settlement has not been investigated adequately.

To address these issues, this study compares three optimisation algorithms, i.e. the butterfly optimisation algorithm (BOA), sparrow search algorithm (SSA), and slime mould algorithm (SMA). The selection of the SMA to optimize the hyperparameters of the Random Forest (RF) is considered optimal. The rationality of the dimension reduction is validated by considering the feature contribution and Spearman correlation coefficient. A double-input deep neural network (D-DNN) framework is constructed while considering the effects of double-tunnel excavation and increasing the database capacity. The framework comprises two branches that receive input data from the left and right lines of shield tunnelling. Data from monitoring points at the surface monitoring levels (levels I and II) of Jinan Metro Line 4 are obtained to evaluate the performance of the D-DNN for training and prediction. A comparison is performed using a single-input deep neural network (S-DNN) framework, and the prediction results are evaluated using the coefficient of determination (R^2^) and mean absolute error (MAE). The outline of the study consists of six sections, which are as follows:

1. Introduction
2. Methodology
3. Database
4. Feature selection and analysis
5. Analysis of predicted results
6. Conclusion

## Methodology

### RF

**Figure 1:**
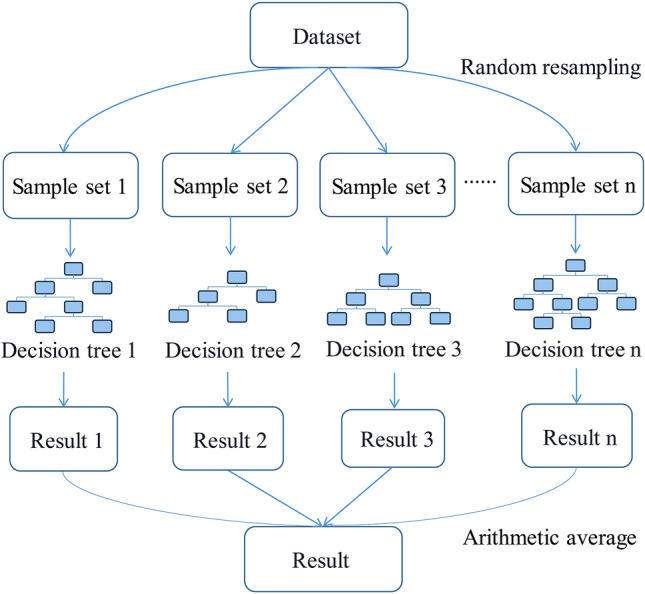
Principle diagram of RF.

An RF^47^ is an ensemble algorithm that integrates cart decision trees via a bagging algorithm and can address both classification and regression problems. The calculation process of an RF is shown in Fig. 1. Samples and features are randomly sampled by the RF, and the optimal feature in the random samples is selected to segment the cart decision tree into left and right subtrees, thus forming different cart decision trees. In the prediction and regression process, each decision tree analyses and predicts the input parameters, and the arithmetic average of the regression results obtained by weak cart learners is calculated to obtain the final model output.

### Optimistic algorithm

#### BOA

The BOA was proposed by Arora and Singh^48^ based on the foraging and mating behaviours of butterflies. The scent emitted by butterflies is correlated with their adaptability levels. Butterflies function as search agents and are attracted to the butterflies with the strongest scent, thus enabling a global search. In the absence of scent perception by other butterflies, they engage in random movements to perform a local search. The intensity of the butterfly scent depends on physical stimulation, which can be expressed as

1 $$ f = cI^{\alpha } $$

where *f* represents the odour coefficient, *c* the sensory factor, *I* the stimulation intensity related to fitness, and *a* the power exponent.

#### SSA

The SSA was proposed by Xue and Shen^49^ based on the foraging and anti-predatory behaviours of discoverers and joiners in a sparrow population. Discoverers, joiners, and vigilant individuals are present in a sparrow population. Discoverers have higher energy reserves and are tasked with identifying food-abundant areas. Their fitness level is high, which can provide a foraging direction and area for the joiners. Joiners follow discoverers, monitor them, and compete for resources. When sparrows in the population sense danger, the edge sparrows of the group propagate promptly to a safe area to obtain a better position. The sparrows in the middle of the population randomly propagates closer to the other sparrows to reduce the probability of being preyed on.

#### SMA

The SMA is a new type of metaheuristic algorithm proposed by Li et al.^50^. It achieves intelligent optimisation by simulating the foraging behaviour of slime moulds as they approach, surround, and capture food. The algorithm generates a random group of individual slime molds. Each slime mold adjusts its cytoplasmic concentration in response to the food concentration, enabling them to move towards higher concentrations of food. By employing iterative optimization, the algorithm ultimately discovers the optimal solution. The entire process is executed using functional expressions, and further details can be found in reference^50^.

### D-DNN

**Figure 2:**
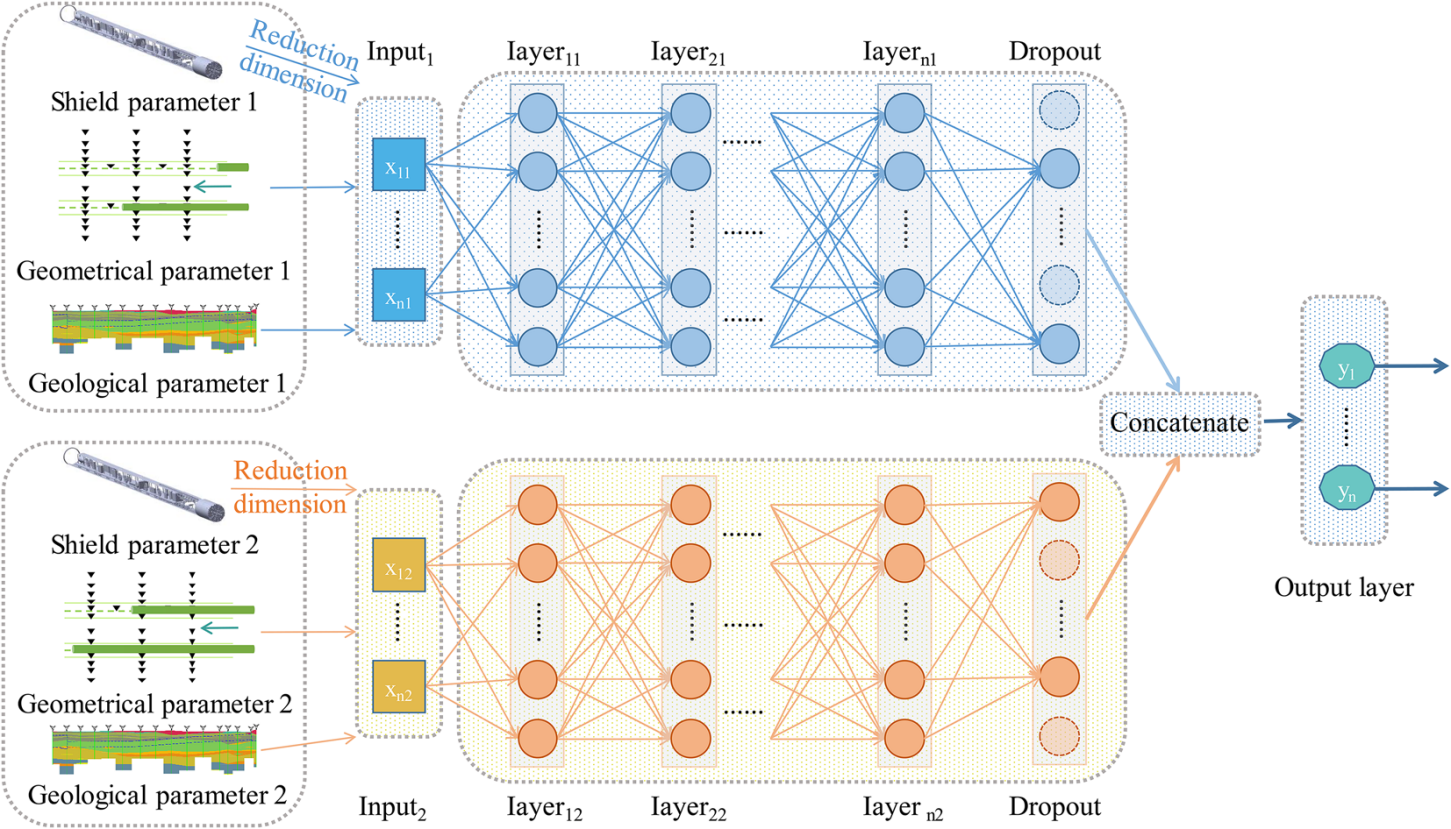
D-DNN framework.

DNN comprises multiple layers of neurons, with each layer fully connected to the next layer. The input layer receives signals, the hidden layer extracts features, and the output layer predicts the results. Each layer of neurons comprises a bias term and a set of weights^51^. The output result is obtained by computing the weighted sum of the input signals and weights, considering the bias term, and then passing through an activation function. During double-line tunnel excavation, surface settlement is caused by the simultaneous operation of two shield machines. Surface settlement is affected by the spatial position of the two shield machines and their respective parameters. The accurate allocation of the respective parameters of both shield machines is crucial for investigating the longitudinal surface settlement. The D-DNN framework, as shown in Fig. 2, comprises two branches: Input_1_ and Input_2_. Each branch uses the shield tunnel excavation parameters after dimension reduction, the geometric parameters, and the geological parameters as inputs. Upon inputting two sets of parameters related to the shield machine into the input layer, the hidden layer extracts the features from the input. Additionally, the backpropagation algorithm is continuously utilised to adjust the weights of each individual feature, thus resulting in the generation of feature vectors. Dropout layers are added at the end of the neural network layers in both branches to prevent overfitting and ensure regularisation. After feature extraction, the feature vectors extracted from both tunnels are merged and connected to the output layer to generate the final output.

**Figure 3:**
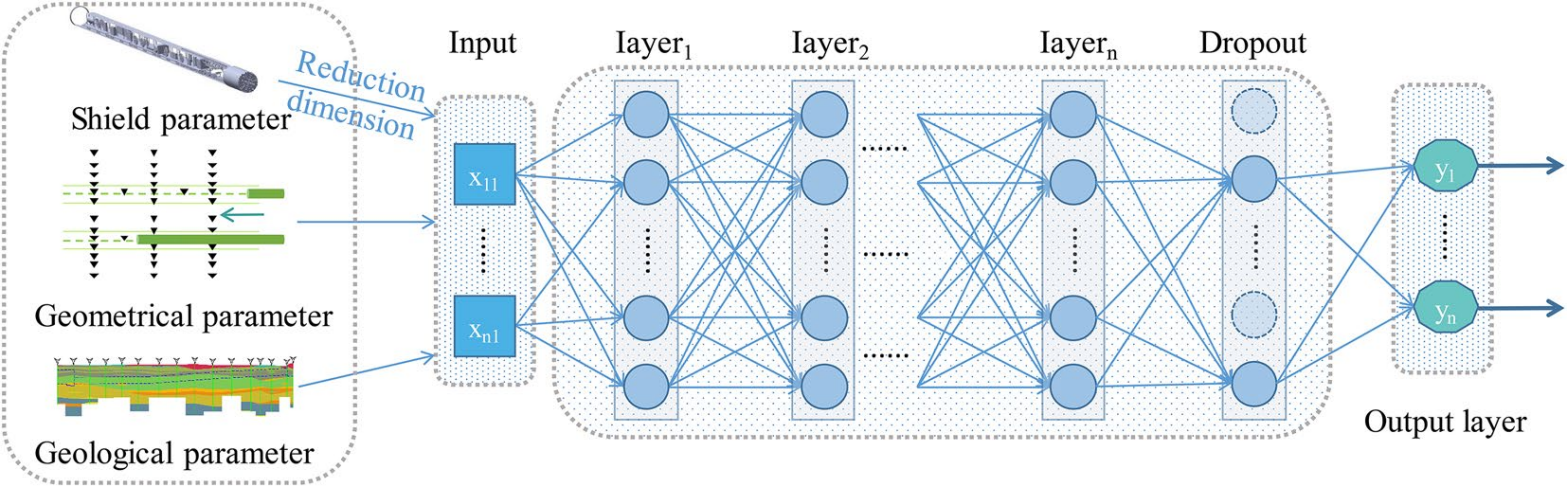
S-DNN framework.

In this study, the performance of the D-DNN will be evaluated by comparing it with the S-DNN which is a general modelling framework^52,53^. The specific structure of the S-DNN can be seen in Fig. 3. Similar to the D-DNN, the S-DNN deals with situations where there is only one input path. In this case, the input consists of parameters related to shield operational parameters, geometric parameters, and geological parameters of a single tunnel. The backpropagation algorithm is continuously applied to calculate the weights of each input feature during the training process. These weights are then directly connected to the output layer to generate the final results. Since the S-DNN model only takes into account the input parameters related to a single tunnel, it has a single input path. Consequently, the training process does not involve merging feature vectors.

### Method flowchart

**Figure 4:**
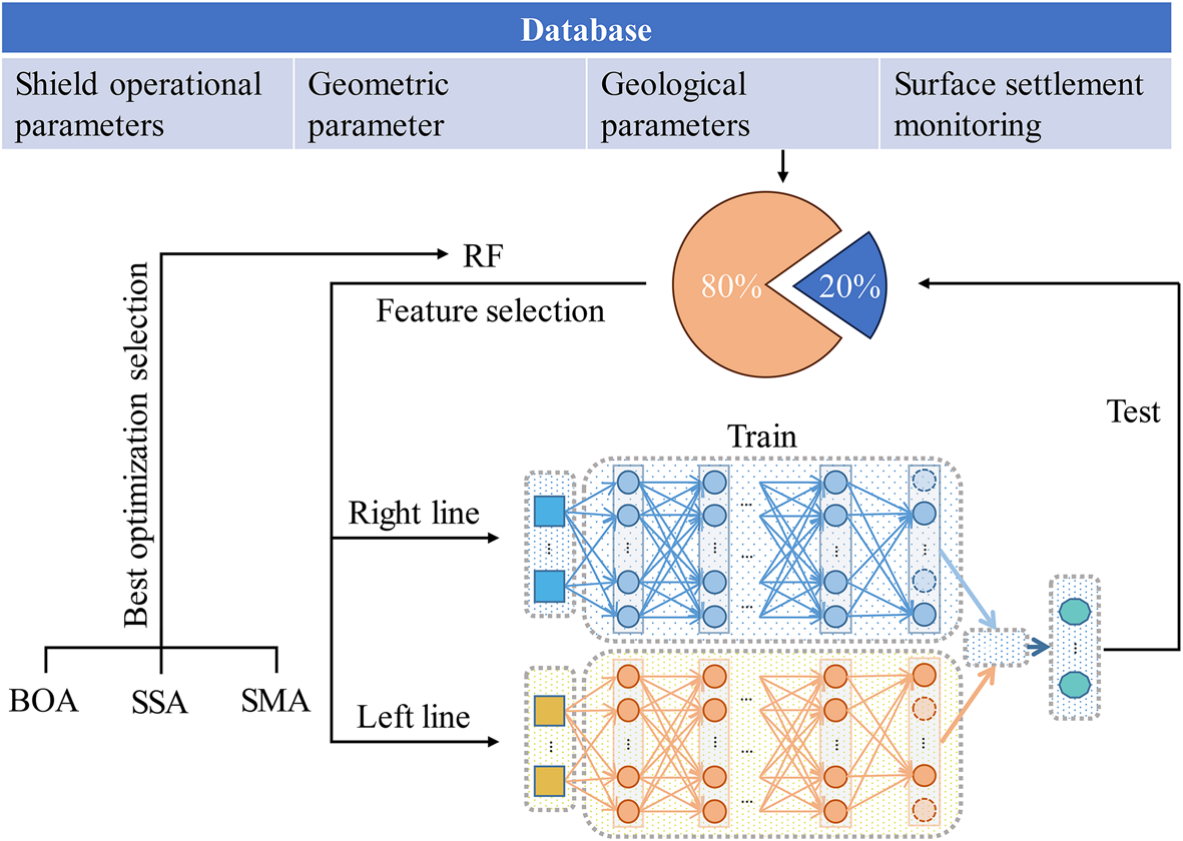
Method flowchart.

The flowchart in Fig. 4 illustrates the process of training and testing longitudinal surface settlement. In the databases of the left and right lines, the optimized RF is used to analyse the feature contribution and perform feature selection on 80% of the dataset. The purpose of doing so is to ensure the confidentiality of data information during feature selection and prevent data leakage to the testing set. Afterwards, the selected features from the left and right lines are used as input for the D-DNN framework to train and obtain the weight relationship between input features and surface settlement. Finally, these weight values are used to test the model's performance on the remaining 20% of the data for performance evaluation.

### Performance indicators

The predicted results were obtained after inputting the features into the D-DNN and S-DNN models. In consideration of the potential for biased estimates when using a single R^2^ indicator^54^, we use both R^2^ and MAE indicators to collectively evaluate the performance of the model’s predictions. R^2^ assumes values in the range [0,1], where a higher value indicates better prediction performance, whereas MAE assumes values in the range [0,∞), where a lower value indicates better prediction performance. The R^2^ and MAE are calculated as follows:

2 $$ {\text{R}}^{{2}} = 1 - \frac{{\sum\nolimits_{i = 1}^{n} {\left( {y_{i} - y^{\prime}_{i} } \right)^{2} } }}{{\sum\nolimits_{i = 1}^{n} {\left( {y_{i} - \overline{y}} \right)^{2} } }} $$

3 $$ {\text{MAE}} = \frac{1}{n}\left( {\sum\nolimits_{i = 1}^{n} {\left| {y_{i} - y^{\prime}_{i} } \right|} } \right) $$

where *n* is the number of samples, *y*_*i*_ the* i*th actual value, *y*′_*i*_ the *i*th predicted value, and $\overline{y}$ the average of the actual values.

## Database

**Figure 5:**
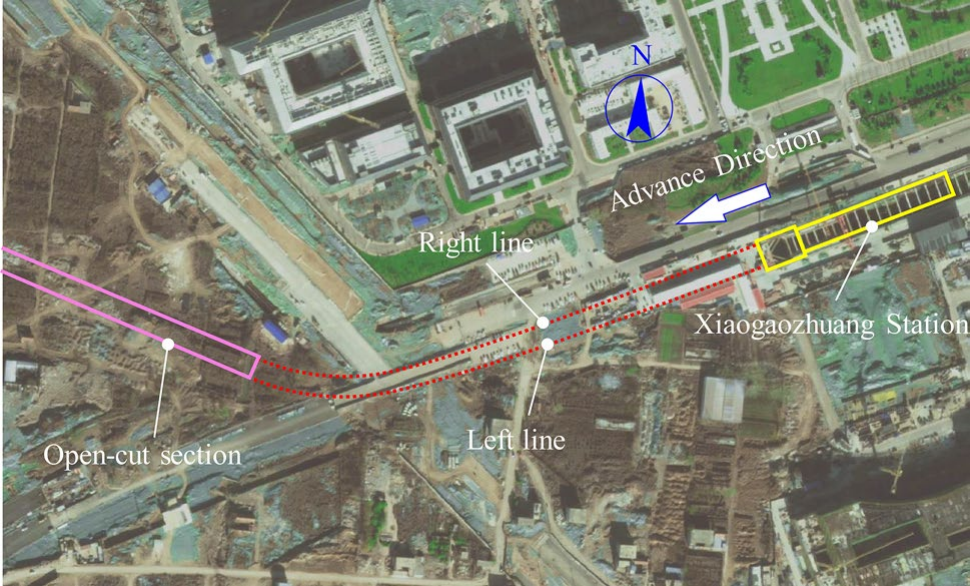
Plan view of construction site.

The research database was obtained from a section spanning approximately 450 m between Xiaogaozhuang Station and Weilizhuang Station on the Jinan Metro Line 4. In this section, two tunnel boring machines were employed for construction, which resulted in the completion of 376 and 371 rings spanning 6.68 m on the left and right lines, respectively. The bottom and top plates of the underground shield structure were buried at depths of 11.80–19.50 and 2.40–13.10 m, respectively. The outer diameter of the pipe segment was 6.40 m, and its ring width of 0.30 m. The obtained data primarily included parameters related to the shield excavation, geometry, geology, and surface settlement and its statistical parameters are shown in Table 2. The construction site plan for this section is illustrated in Fig. 5.

**Table 2:** Statistical parameters of Collecting on-site database.

Variable type	Variable (orientation)	Left line					Right line					Units
		Min	Max	Ave	Sd	Ske	Min	Max	Ave	Sd	Ske	
Shield operational parameters	Cutter head speed	0.02	0.70	0.20	0.13	1.09	0.03	0.80	0.26	0.16	1.12	rpm
	Torque	− 582.15	588.44	7.23	272.49	− 0.14	− 1124.95	821.39	− 21.11	421.91	− 0.12	kN·m
	Chamber pressure (top-left, bottom-left, mid-left, top-right, bottom-right)	0.16	2.65	1.43	0.49	− 0.28	0.00	2.05	1.41	0.43	− 1.50	bar
	Thrust (top, bottom, left, right)	3.66	261.92	68.51	44.16	1.19	11.62	236.85	89.94	41.62	0.88	bar
	The time used for each ring	1.51	136.76	13.37	23.42	3.24	0.86	84.03	9.43	16.50	2.95	h
	Bentonite pressure	0.00	1.72	0.48	0.47	1.04	0.02	1.87	0.31	0.41	2.05	bar
	Grouting pressure (top-left, bottom-left, mid- left, top-right, bottom-right)	0.00	1.92	0.37	0.50	1.33	0.00	3.35	1.94	0.90	− 0.77	bar
	Hinged displacement (top-left, bottom-left, mid- left, top-right, bottom-right)	23.03	118.86	58.31	19.00	1.23	31.22	95.16	53.05	13.49	1.33	mm
	HBW seal pressure (internal, external)	0.22	2.00	1.45	0.37	− 0.97	0.01	2.18	1.63	0.42	− 2.21	bar
	Thrust speed	0.02	11.49	5.07	3.21	− 0.12	0.00	19.58	6.49	4.80	0.62	mm/min
	Screw speed	0.00	1.58	0.75	0.46	− 0.13	0.00	2.62	1.02	0.72	0.42	rpm
Geometric parameter	Overlying soil thickness	1.20	380.40	96.67	125.64	1.12	2.40	382.80	72.49	105.61	1.71	m
	Distance separating the monitoring point from the shield tunnel ring	0.00	391.20	104.90	130.94	1.05	1.20	393.60	74.49	108.53	1.73	m
Geological parameters	Weighted average* E*_*s*_	5.32	6.26	5.83	0.19	− 0.28	5.25	6.28	5.83	0.22	− 0.39	MPa
Surface settlement	Settlement	− 23.86 (Min)		1.18 (Max)		− 9.35 (Ave)		9.16 (Sd)		− 0.30 (Ske)		m

Minimum (Min); Maximum (Max); average (Ave); Standard deviation (Sd); Skewness (Ske).

### Shield operational parameters

The shield tunnelling parameters were monitored from an intelligent platform that can monitor parameters such as the thrust pressure, chamber earth pressure, and amount of bentonite used. Each parameter was monitored and recorded once every second. The excavation data of the shield tunnelling machines from the left and right tunnels were obtained. The values of various parameters were averaged based on the shield rings, which resulted in 91 characteristic quantities for each tunnel and a combined total of 182. To ensure that the input parameters contributed to the objective function and to avoid data information loss, the values of the shield operational parameters obtained were not summed. For example, the grouting pressure included the grouting pressure in four directions: top-left, top-right, bottom-left, and bottom-right, which were counted as four input features. Table 2 shows some of the value of the excavation parameters obtained.

### Geometric parameters

The research object was longitudinal surface settlement, and the related geometric parameters were the tunnel diameter, overlying soil thickness, and distance between the monitoring point and shield tunnel ring. In the case of shield tunnel excavation in engineering, where a constant diameter is used, we were unable to obtain valuable information regarding surface settlement. Consequently, this factor was excluded as a feature input in the present study. The selected geometric parameters encompassed the overlying soil thickness and the distance separating the monitoring point from the shield tunnel ring.

### Geological parameters

The shield tunnel segment is situated in the alluvial plain of the Xiaoqing River, which is a tributary of the Yellow River characterised by a predominantly flat terrain. Based on the drilling data, the predominant soil types in this area included silty clay, clay, fine sand, and gravel. The challenges identified were associated with the selection and quantification of geological parameters. Hussaine and Mu^55^ used soil type as an input parameter, whereas Su et al.^56^ quantified the internal friction angle and cohesive force using the weighted averaging method. To mitigate overfitting, only a limited set of significant parameters should be used as input features. Yuan et al.^57^ investigated the relationship between soil parameters and surface settlement and concluded that the compression modulus (Es) exerted the most prominent effect on surface settlement.

Because the tunnels in this project primarily passed through layered silty clay and fine sand and the soil type of the tunnel excavation face was relatively constant, the soil type was not used as an input. The weighted average Es was the only input among the geological parameters. *Es* can be calculated as follows:

4 $$ E_{s} = \sum\nolimits_{i}^{n} {E_{si} } \cdot h_{i} $$

where *E*_*si*_ is the compression modulus of the *i*th soil layer (MPa), *h*_*i*_ the thickness of the *i*th soil layer (m), and *n* the total number of soil layers above the bottom of the tunnel.

**Figure 6:**
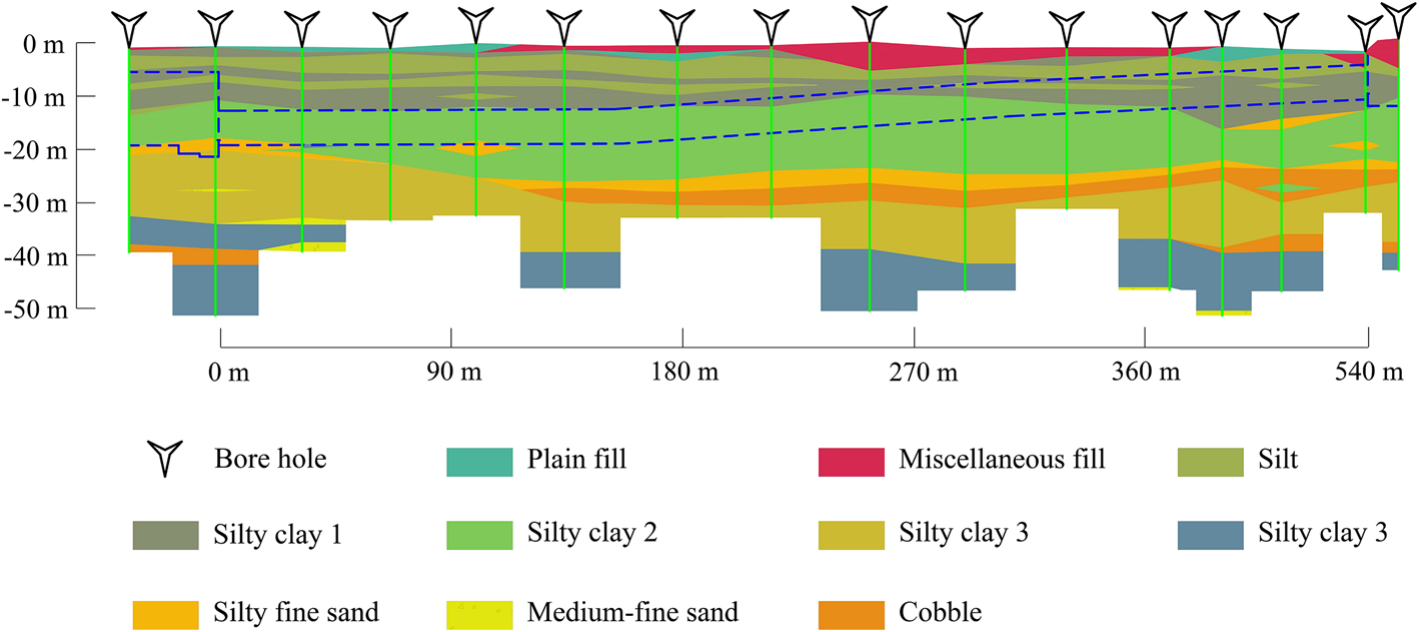
Geological cross-section of study area.

The geological section diagram of the study area is shown in Fig. 6.

### Surface settlement monitoring

**Figure 7:**
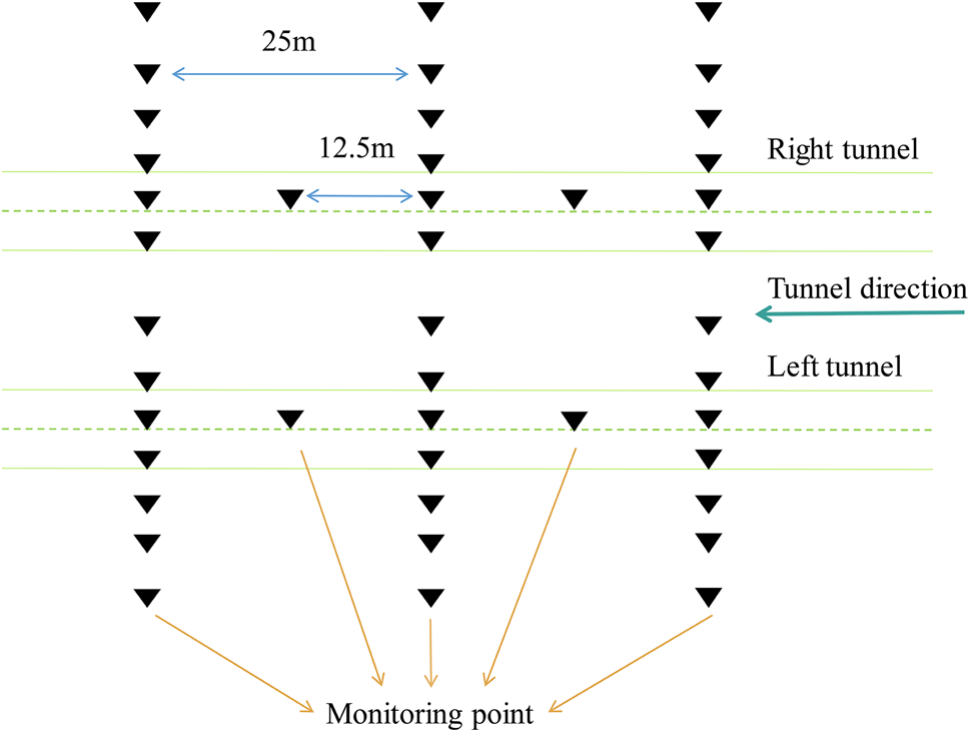
Schematic diagram of surface settlement monitoring point layout.

Surface settlement monitoring points were arranged above and around the shield tunnel to monitor the effect of shield excavation on the surrounding environment. A schematic illustration of the surface settlement monitoring point layout is shown in Fig. 7. Along the tunnel centreline, a monitoring point was arranged every 12.5 m and a monitoring section was set every 25 m, with 13 monitoring points arranged on it. In accordance with GB 50911-2013^58^, settlement thresholds of 10–20, 20–30, and 30–40 mm correspond to the Level I, Level II, and Level III monitoring thresholds, respectively. The maximum settlement was 28.7 mm, and no monitoring points had settlement levels reaching Level III. In this study, data from two monitoring points in actual engineering were obtained for training and testing; the monitoring points were DBC-6-1, which reached the Level I monitoring grade; and DBC-7-4, which reached the Level II monitoring grade.

## Feature selection and analysis

The input of the D-DNN included parameters for double-line shield tunnelling, geometric parameters, and geological parameters. However, the parameters for shield tunnelling may contain invalid or redundant data. Including all these inputs will degrade the performance of the neural network and may result in weight learning failure. To select features efficiently and effectively, the optimisation effects of the BOA, SSA, and SMA on an RF were compared, and SMA-RF was selected as the best option for the dimensionality reduction of the high-dimensional features of shield operational parameters. Meanwhile, the rationality of the dimensionality reduction was validated based on two aspects: the feature contribution and Spearman correlation coefficient. Subsequently, the reduced shield operational parameters were combined with the geometric and geological parameters to serve as input for the D-DNN. Using the data from monitoring points DBC-6-1 and DBC-7-4, respectively select the top 80% of each dataset for feature selection, and conduct analysis on feature contribution and correlation.

### Selection of optimisation algorithms

Based on the first 80% of the entire dataset, further divide it by selecting 80% of the data for training and keeping the remaining 20% for testing. Three optimisation algorithms, BOA, SSA, and SMA, were used in this study to optimise the hyperparameters of an RF, including the number of trees in the forest (n_estimators) and the maximum depth of the tree (max_depth). The mean squared error was used as the fitness function (cost function), where a smaller fitness value corresponds to a better optimisation result. Because optimisation algorithms involve random numbers, when the same optimisation algorithm is used to optimise the same problem multiple times, the results of each optimisation will differ slightly. Therefore, multiple experiments were performed, and the mean values were calculated for evaluation. To ensure fairness between the three algorithms, the same parameters were set for each experiment: 30 trials, 50 population sizes, and a maximum of 200 iterations, n_estimators ∈ [1,200], max_depth ∈ [1,20].

**Figure 8:**
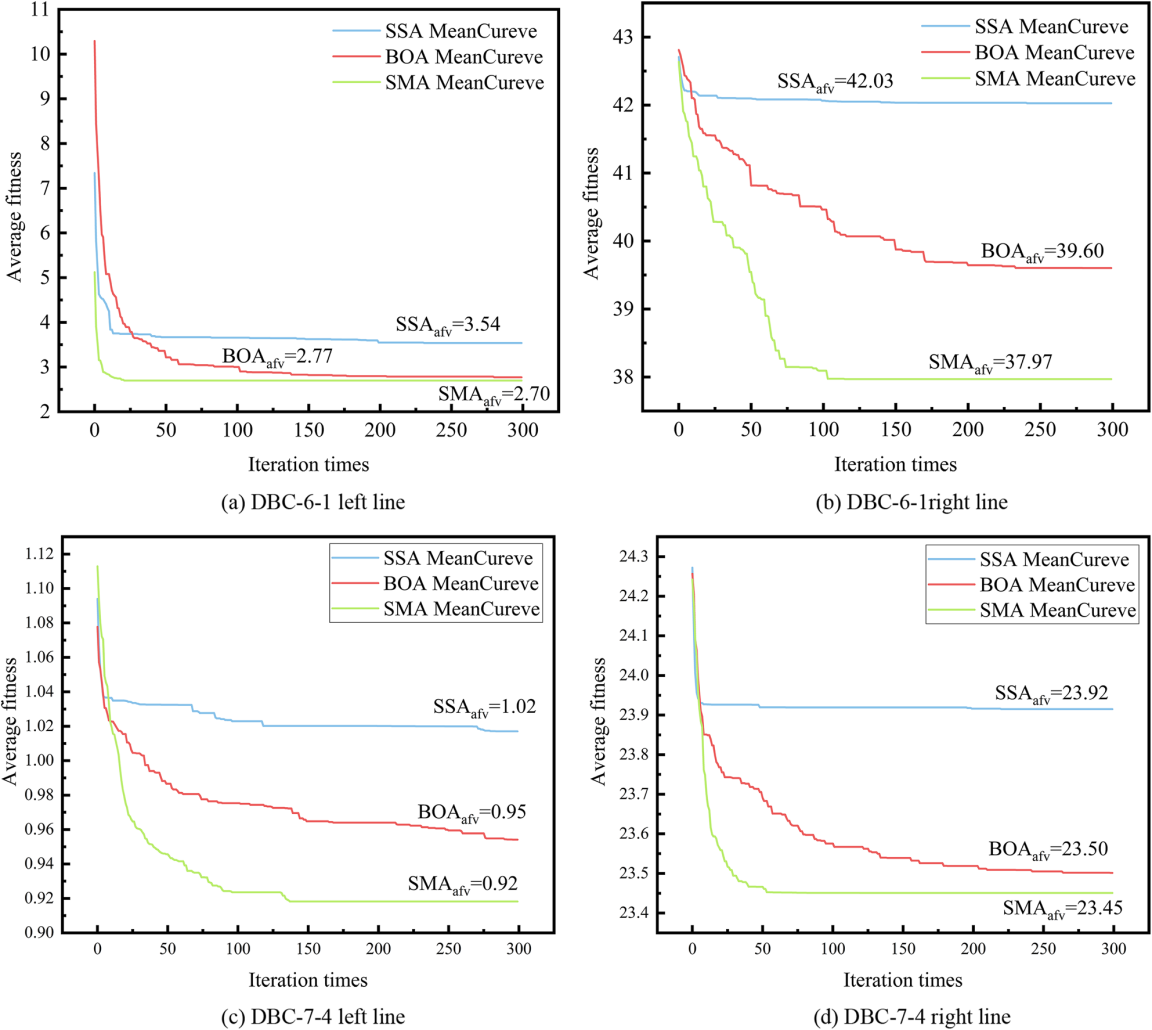
Average fitness curves of the three algorithms for optimising left and right lines.

Because the deep learning model is a double-input model, the left and right twin tunnels were optimised separately. The average fitness curves of the three selected optimisation algorithms are shown in Fig. 8.

Figure 8a, b demonstrate that SMA performs better than SSA and BOA in terms of search speed and final average fitness value. SSA converges prematurely, while BOA performs better in solving the average fitness value, but there is a certain gap between SMA in terms of search speed and the lowest average fitness value. The Fig. 8c, d indicate an initial lag of SMA compared to BOA and SSA in terms of search speed. However, as the iteration process proceeds, after approximately 10 iterations, its optimization speed becomes the best, ultimately reaching the lowest average fitness value. Figures 7d and 8c reveal that SMA initially lags behind BOA and SSA in terms of search speed. However, as the iteration process progresses, its optimization speed gradually becomes the best, ultimately achieving the lowest average fitness value. SSA and BOA demonstrate similar patterns, as illustrated in Fig. 8a, b. In conclusion, SMA performs the best in the optimization of RF's hyperparameters in the data of DBC-6-1 and DBC-7-4. SSA has the issue of early convergence, and BOA's performance lies between the SSA and SMA. Consequently, we select SMA as the method for optimizing the hyperparameters of RF. Table 3 presents the results of RF parameter optimization achieved using SMA.

**Table 3:** SMA optimisation results.

Hyperparameter	DBC-6-1		DBC-7-4	
	Left line	Right line	Left line	Right line
n_estimators	1	1	6	7
max_depth	5	6	8	7

### Results of dimensionality reduction using SMA-RF

**Figure 9:**
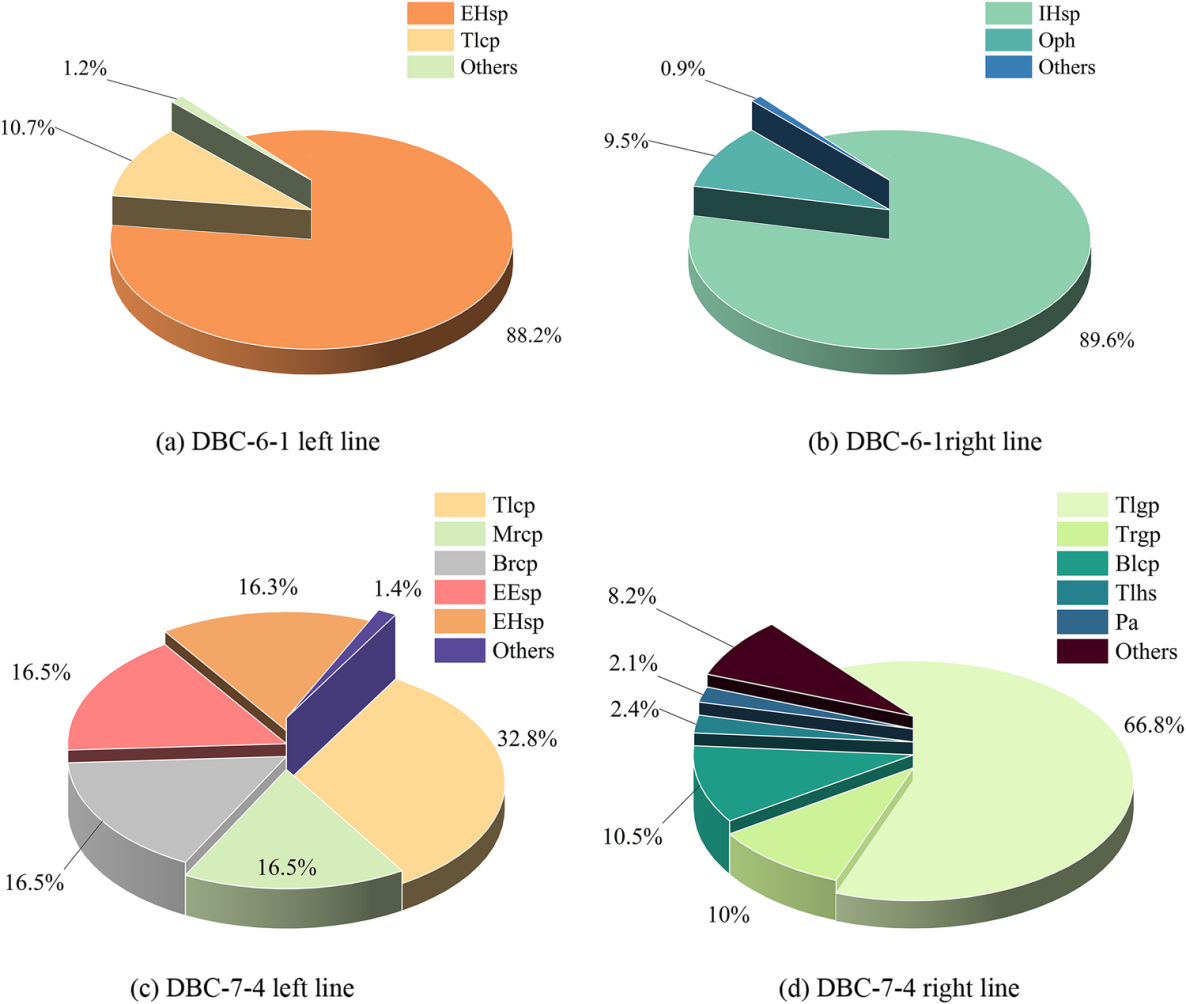
Contributions of shield operational parameters of left and right lines.

SMA-RF was utilised to analyse the contribution of the tunnelling parameters to the features of the shield in the left and right lines separately. Features that contributed significantly were selected as inputs to the D-DNN. As shown in Fig. 9, the contribution of the shield machines to the surface settlement differed between the left and right lines, and the contribution proportions of each feature were uneven. For example, in Fig. 9a, the external HBW seal pressure (EHsp) on the left line presented a significant contribution ratio of 88.2%, whereas the top-left chamber pressure (Tlcp) contributed 10.7%. Meanwhile, the remaining 89 features collectively contributed only 1.2% of the overall contribution.

The above results indicate that in the case of small sample data, the contribution proportions and distribution of each feature are uneven, leading to significant uncertainties in machine learning tasks. The potential of each feature to contribute in subsequent training and prediction models will also vary. Therefore, unlike the feature selection in existing research^27,59,60^, considering that feature selection is based on the selection from the entire database, with a large number of features (182 features), and also considering the high uncertainty in small sample data. We might as well adopt the idea of specific analysis for specific problems^31^, focusing on the features that have great potential for deep learning tasks. In this study, we select features with a cumulative contribution value greater than 90% as input, aiming to obtain a model with good test performance while avoiding an excessive number of input features. In the DBC-6-1, the left line uses EHsp and Tlcp as input features, while the right line uses the internal HBW seal pressure (IHsp) and the outlet pressure of the hinged pump (Oph) as input features. In the DBC-7-4, the left line uses top-left chamber pressure (Tlcp), middle-right chamber pressure (Mrcp), bottom-right chamber pressure (Brcp), the external EP2 seal pressure (EEsp), and the external HBW seal pressure (EHsp) as input features, while the right line uses top-left grouting pressure (Tlgp), top-right grouting pressure (Trgp), bottom-left chamber pressure (Blcp), top-left hinged displacement (Tlhs), and pitching angle (Pa) as input features.

### Spearman correlation analysis

In order to further analyse the features that contribute to 90% of the cumulative feature contribution, taking DBC-6-1 as an example, the Spearman correlation coefficient was analysed to determine the correlation between the selected features on the left and right lines and the surface settlement. The feature variables were organised, and the Spearman correlation coefficient for each feature was calculated as follows:

5 $$ \rho = 1 - \frac{{6\sum {d_{i}^{2} } }}{{n\left( {n^{2} - 1} \right)}} $$

where *d*_*i*_ is the difference between the rank order values of the* i*th data pair, and* n* is the total number of observed samples.

**Figure 10:**
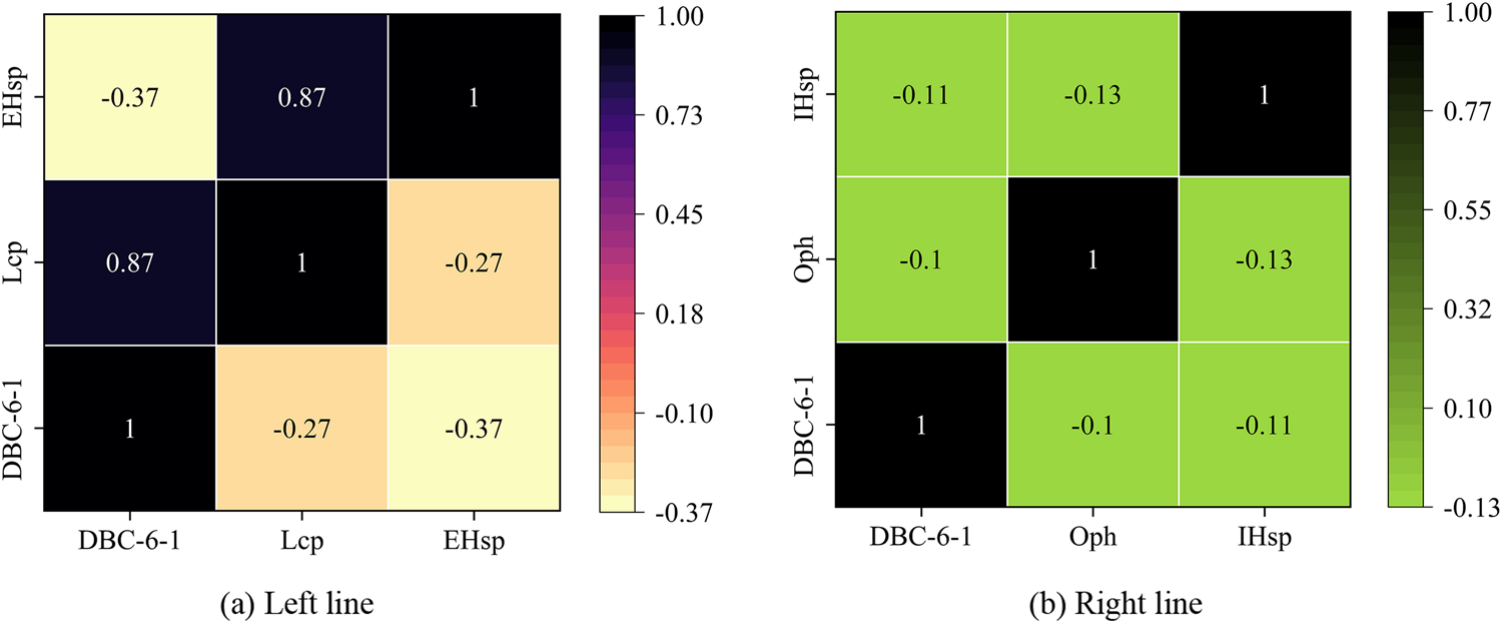
Heat map of spearman correlation coefficient on left and right lines.

The heat maps of the calculated correlation coefficients are shown in Fig. 10. The reduced characteristics of the left and right SMF-RF lines exhibited a weak correlation with the settlement, thus suggesting that the surface settlement was affected by various characteristics, including the shield operational parameters, geometric parameters, and geological factors. The individual characteristics of the shield operational parameters exhibited a high level of nonlinearity with the surface settlement.

Figure 10a illustrates the negative correlation between EHsp and Tlcp and surface settlement. Similar to the findings of a previous study^61^, a higher pressure resulted in reduced surface settlement. The correlation coefficient between EHsp and Tlcp was 0.87, thus indicating a positive correlation. A higher chamber pressure corresponds to an increased EHsp owing to the role of the outer sealing of HBW in isolating mud from water in the chamber. The sealing cavity of the HBW was connected to the chamber, and the sealing pressure exceeded the chamber pressure. Figure 10b shows the negative correlation between IHsp and Oph. The correlation between the two variables was weak, where higher values were associated with reduced surface settlement.

**Figure 11:**
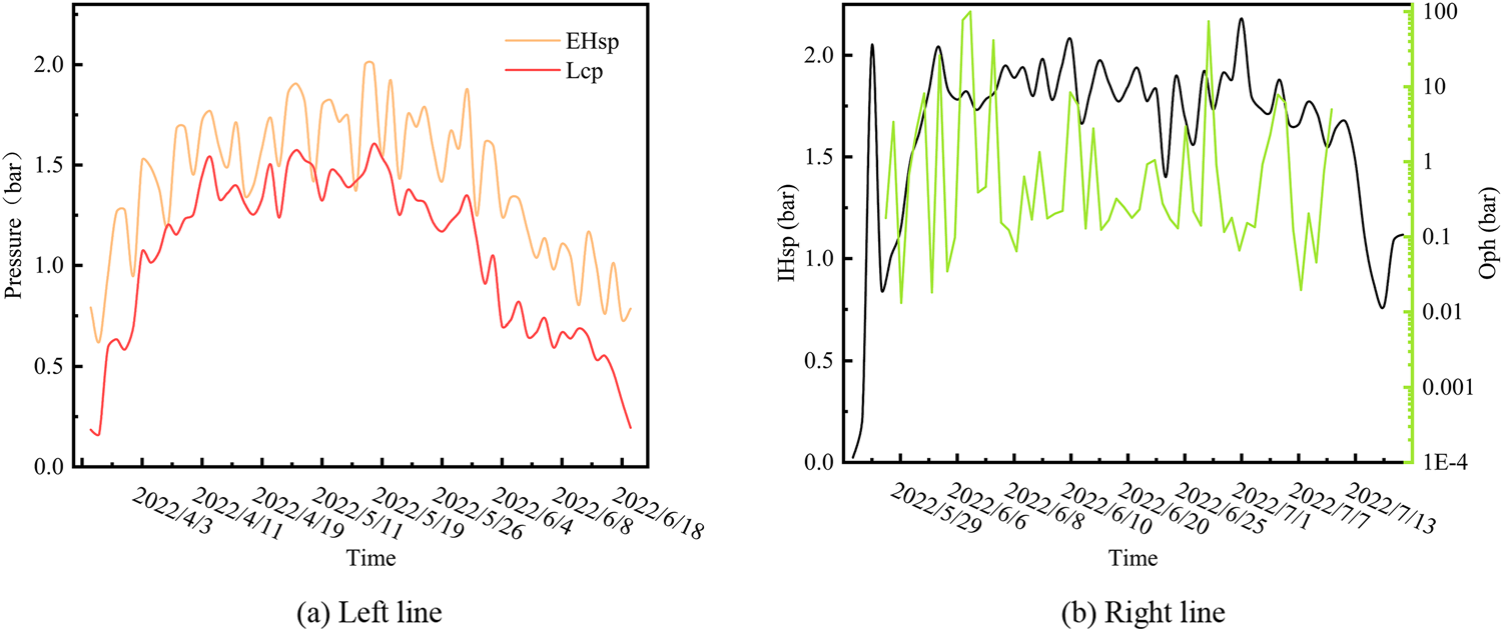
Trend charts of selected features on left and right lines.

Figure 11 illustrates the temporal variations in the reduced-dimensional features. Figure 11a shows a high level of similarity between the variation trends of EHsp and Tlcp. The two variables exhibited a strong positive correlation, with EHsp generally exhibiting higher values than Tlcp. This suggests the effectiveness of the external sealing of the HBW and the intended operation of the shield machine’s mud and water prevention mechanisms. As shown in Fig. 11b, a logarithmic coordinate system was employed to represent the wide-ranging values of Oph. The changing trends of the two feature variables did not indicate similarity; in fact, the trend and value size differed significantly, thus confirming the weak correlation between the two features obtained from Fig. 10b.

In summary, the shield operational parameters are highly nonlinearly related to the surface settlement, which is jointly determined by the shield operational, geometric, and geological parameters. Among the cumulative feature contributions of the top 90% features, there still exists significant correlation among the features. Depending on the complexity of the target task, it can be considered whether to further reduce the dimensionality using methods such as principal component analysis. Considering that the selected features are sufficient to meet the surface subsidence prediction task, this study will no longer reduce the dimensionality further.

## Analysis of predicted results

**Figure 12:**
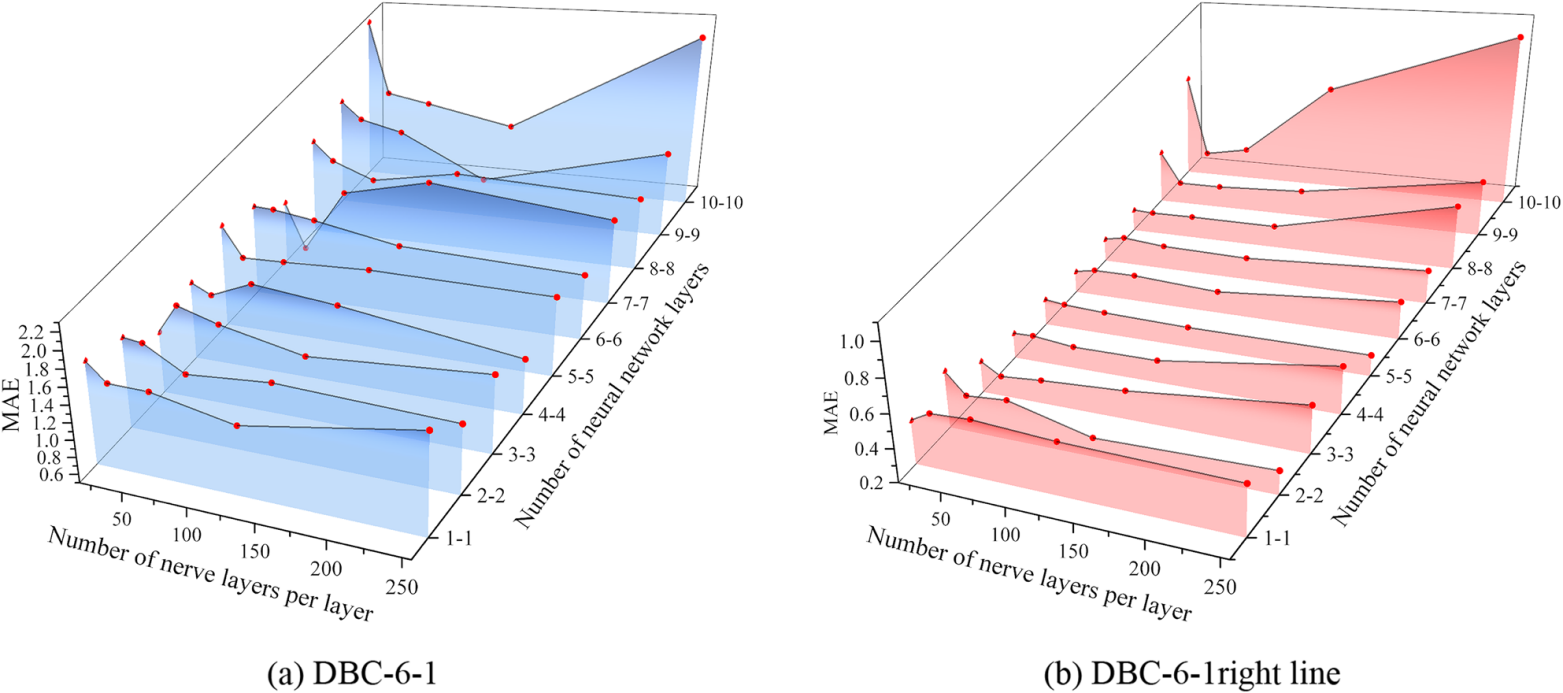
Scatter plots of training and testing sets for S-DNN and D-DNN.

To find the optimal structure of the D-DNN framework, the modelling of monitoring points DBC-6-1 and DBC-7-4 was carried out separately. Based on 80% of the total database, further division was conducted, with 80% of the data chosen for training and the remaining 20% for validation, ensuring that the divided data is completely consistent with the data used in Section "Selection of optimisation algorithms". After training the model, the MAE was used as the cost function to evaluate the performance of the structure in the validation set. A smaller MAE value indicates better structural performance. Figure 12 shows the performance of D-DNN under different structures. It can be observed that, with the number of neurons increasing while keeping the neural network layers constant, the MAE generally shows a decreasing trend followed by an increase. Similarly, keeping the number of neurons constant, an increase in the number of neural network layers shows a similar trend in the MAE value. This is because an excessive number of neurons and neural network layers may lead to overfitting, whereas the opposite may lead to underfitting. Through comparison, the optimal framework structure chosen in the end is as follows: two input paths of DBC-6-1 each have 2 layers of neural layers, with each layer containing 32 neurons; two input paths of DBC-7-4 each have 5 layers of neural layers, with each layer containing 128 neurons.

**Figure 13:**
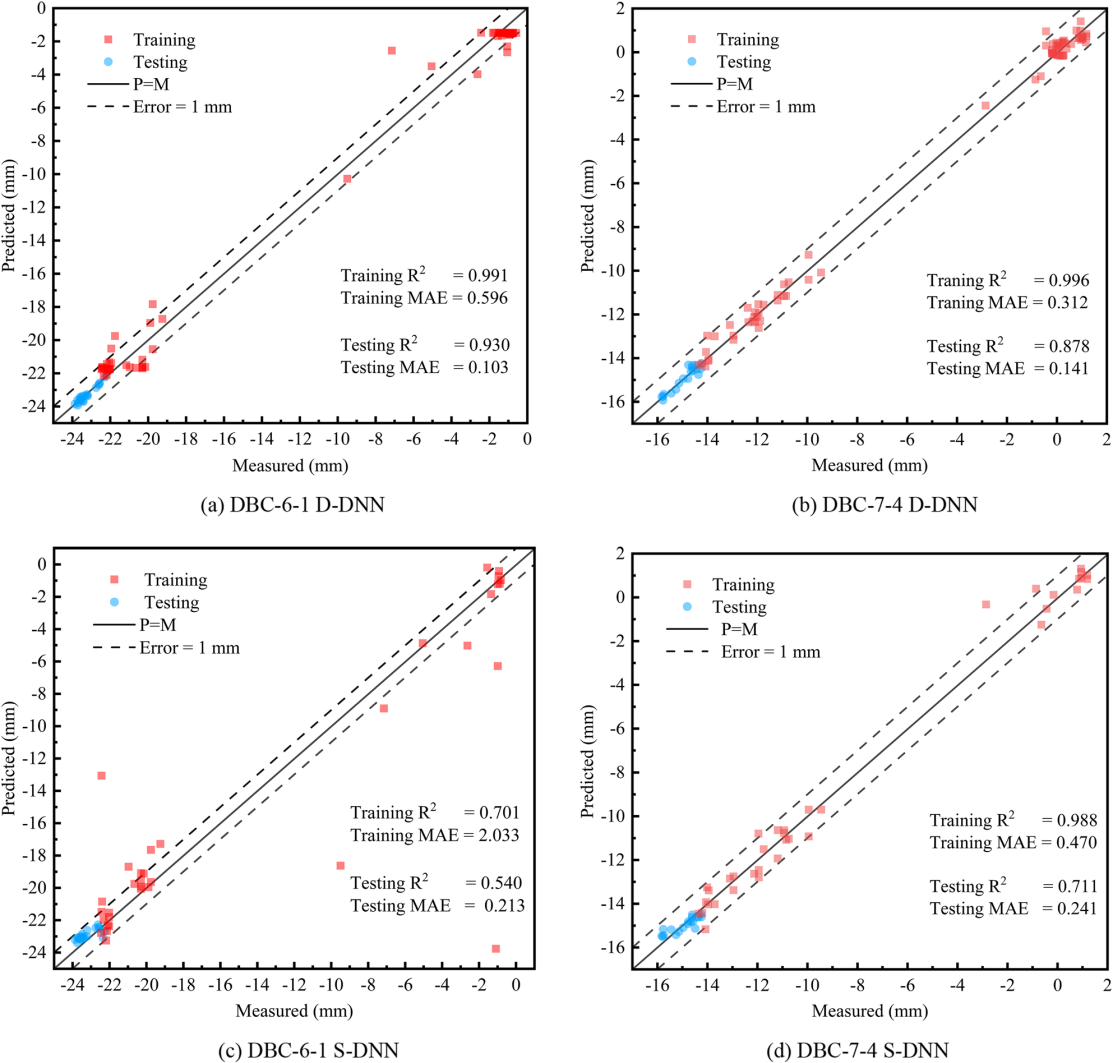
Scatter plots of training and testing sets for S-DNN and D-DNN.

After selecting the best framework structure, the remaining 20% of the overall database is used for prediction, and the prediction results are compared with S-DNN (DBC-6-1, 3 layers of neural layers, 128 neurons per layer; DBC-7-4, 3 layers of neural layers, 64 neurons per layer). The scatter plots for the training and testing sets based on the D-DNN and S-DNN are shown in Fig. 13. Figure 13a, b illustrate the prediction results obtained using the D-DNN. The performance of the D-DNN, which considers double-tunnel boring in shield excavation, is noteworthy in both the training and testing phases. Most of the training set data were within the 1 mm error margin, whereas all R^2^ values of the testing set exceeded 0.85. Moreover, the MAE values were relatively low, thus indicating an acceptable prediction error. Figure 13c, d show the prediction results yielded by the S-DNN. Compared with the results of the D-DNN, the scatter plots for the S-DNN showed fewer data points, and both the training and testing sets showed greater scattering. Furthermore, the test results indicate that the performance of D-DNN surpasses that of S-DNN, with an average increase of 27.85% in R^2^ and an average reduction of 53.2% in MAE.

The differences in the prediction results between the two frameworks were primarily attributed to the following reasons: (1) Different data volumes—the D-DNN fully utilises data from both tunnels, with a data volume exceeding 1.5 times that of the S-DNN. When a relatively large data volume is used, the relationship between various variables and ground settlement can be investigated more effectively. The effective data volumes for DBC-6-1 and DBC-7-4 are listed in Table 4. (2) The D-DNN considers the characteristics of both the left and right tunnels, which is more consistent with actual engineering compared with the single-line data considered by the S-DNN.

**Table 4:** Number of effective data sets.

Monitoring point	Framework type	
	S-DNN	D-DNN
DBC-6-1	56	101
DBC-7-4	56	95

Effective data refers to dimensionally reduced monitoring point data without missing values.

**Figure 14:**
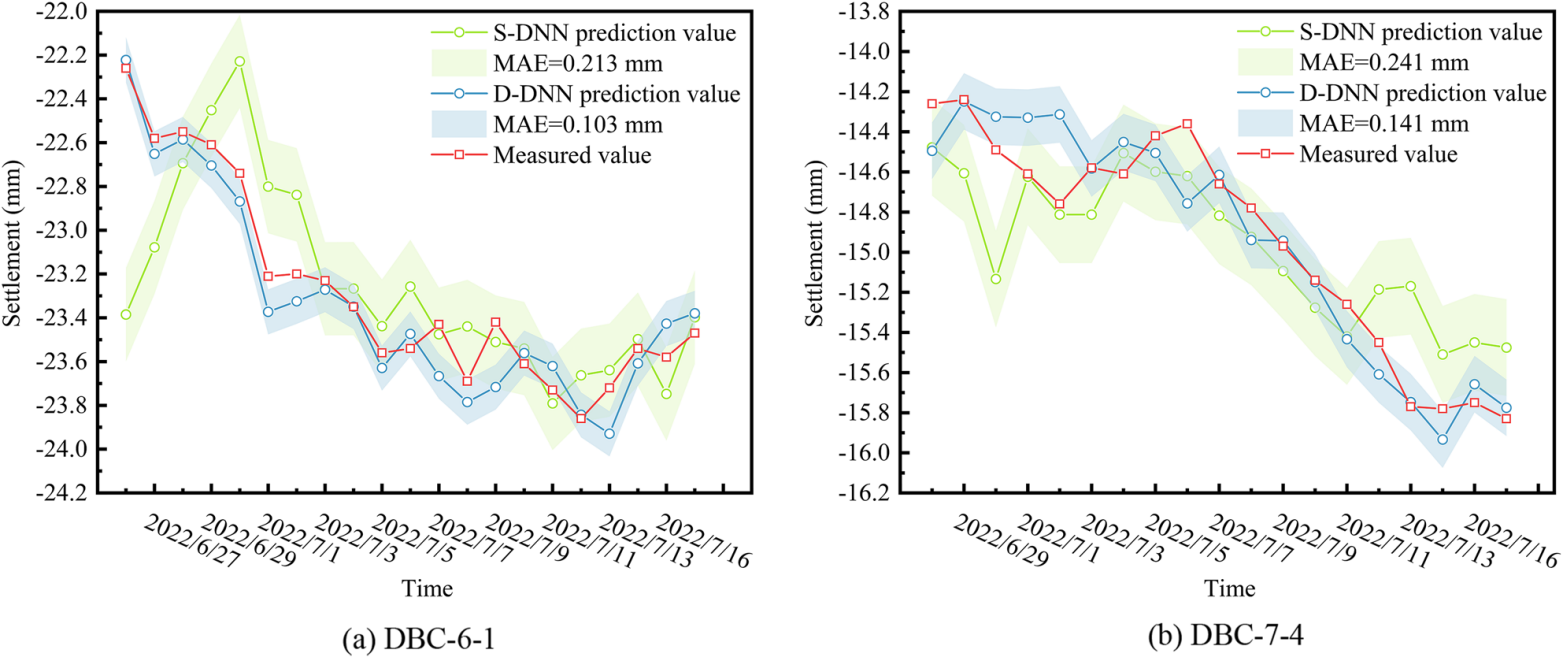
Predicted and measured time-settlement curves.

A comparison of the predicted and measured settlement curves over time is shown in Fig. 14. The values predicted by the D-DNN exhibited excellent performance in terms of both the numerical values and the trend of settlement changes, with only slight deviations from the measured values at the inflection points of the settlement variations. However, the values predicted by the S-DNN deviated significantly from the measured values. The width of the error band for the D-DNN was significantly smaller than that for the S-DNN (see Fig. 12a, b), thus indicating the superior predictive performance of the former. The predicted results indicate that after the tunnel boring machine passed through the monitoring point, the settlement continued to increase; however, the increase was within an acceptable range.

Based on the above analysis, although D-DNN outperforms S-DNN in predictive performance, its framework structure is more complex. In the case of small sample data, optimization algorithms can be used to automate this part of the work in the future.

## Conclusion

To address the problems of small sample data and the excessively high dimensionality of shield operational parameters. In this study, the optimisation effects of three algorithms, namely the BOA, SSA, and SMA, on the hyperparameter optimisation of an RF were investigated. SMA-RF was selected as the optimal algorithm for reducing the dimensions of high-dimensional shield operational parameters. A D-DNN deep-learning prediction method was proposed using the data of left and right shield tunnel lines as inputs to predict the longitudinal surface settlement caused by shield tunnel excavation. Based on the analysis, the following conclusions were obtained:

1. By comparing the optimisation effects of the three algorithms (BOA, SSA, and SMA) on the hyperparameter optimisation of the RF, the results clearly showed that the SMA surpassed both the BOA and SSA in terms of convergence speed and average fitness. The SSA demonstrated premature convergence, whereas the BOA exhibited excellent optimisation capabilities but was not comparable to the SMA in terms of convergence speed.
2. Using the SMA-RF, feature dimension reduction was conducted on the parameters of the double-line tunnel. The prediction results indicated high precision when features with a cumulative contribution exceeding 90% were selected as the input to the D-DNN. The SMA-RF performed well in reducing the dimensions of high-dimensional shield operational parameters.
3. Analysis of features in small sample data leads to increased uncertainty. Contributions of features vary significantly at different monitoring points, as does their potential in deep learning tasks. Thus, in addressing small sample issues, it is advisable to perform targeted analysis of monitoring data under diverse conditions prior to engaging in deep learning tasks to fully utilize existing data.
4. Based on correlation analysis, the relationship between the individual shield operational parameters and surface settlement was shown to be limited, whereas various features of the shield operational parameters exhibited high nonlinearity with surface settlement. The shield operational, geometric, and geological parameters collectively determined the longitudinal surface settlement. There is still a substantial correlation among the selected feature parameters. Depending on the specific context, the need for employing additional dimensionality reduction methods, such as principal component analysis, can be determined.
5. Because it effectively increases the high-fidelity data in the database (more than 1.5 times that of S-DNN) and considers the parameters of the dual-line tunnel, D-DNN exhibits superior training effectiveness and prediction accuracy compared to S-DNN. The test results show that the R2 indicator has improved by an average of 27.85%, while the MAE indicator has decreased by an average of 53.2%. The D-DNN framework proposed in this paper can be applied to predict surface settlement in other shield tunnel projects. For other engineering problems, it is necessary to carry out a re-analysis of feature contributions for feature selection.

This study uses the D-DNN framework to predict longitudinal surface settlement. Despite achieving positive test results with a small sample, the study has the following limitations: (1) When faced with the issue of small data samples, it is advisable to consider using numerical simulations, theoretical formulas, and other methods to augment low-fidelity data while fully utilizing high-fidelity data. This can help reduce the uncertainty of small sample data and achieve better results; (2) the feature selection method is relatively rough, necessitating further research to identify a more suitable approach for small data samples; (3) the study employs a trial-and-error approach to optimize the D-DNN hyperparameters. To achieve full automation, the utilization of optimization algorithms for refining the neural network hyperparameters is warranted. Further research is required in this area.

## Data Availability

The datasets used and/or analysed during the current study are available from the corresponding author on reasonable request.
